# L17A/F19A Substitutions Augment the α-Helicity of β-Amyloid Peptide Discordant Segment

**DOI:** 10.1371/journal.pone.0154327

**Published:** 2016-04-22

**Authors:** Chu-Ting Liang, Hsien-Bin Huang, Chih-Ching Wang, Yi-Ru Chen, Chi-Fon Chang, Ming-Shi Shiao, Yi-Cheng Chen, Ta-Hsien Lin

**Affiliations:** 1 Department of Life Sciences and Institute of Genome Sciences, National Yang-Ming University, Taipei, Taiwan, R.O.C; 2 Basic Research Division, Medical Research Department, Taipei Veterans General Hospital, Taipei, Taiwan, R.O.C; 3 Department of Life Science and the Institute of Molecular Biology, National Chung Cheng University, Chiayi, Taiwan, R.O.C; 4 Structural Biology Program, National Yang-Ming University, Taipei, Taiwan, R.O.C; 5 Institute of Biochemistry & Molecular Biology, National Yang-Ming University, Taipei, Taiwan, R.O.C; 6 Department and Institute of Pharmacology, National Yang-Ming University, Taipei, Taiwan, R.O.C; 7 Genomics Research Center, Academia Sinica, Taipei, Taiwan, R.O.C; 8 Department of Biomedical Sciences, Chang Gung University, Taoyuan, Taiwan, R.O.C; 9 Department of Medicine, Mackay Medical College, Taipei, Taiwan, R.O.C; Russian Academy of Sciences, Institute for Biological Instrumentation, RUSSIAN FEDERATION

## Abstract

β-amyloid peptide (Aβ) aggregation has been thought to be associated with the pathogenesis of Alzheimer’s disease. Recently, we showed that L17A/F19A substitutions may increase the structural stability of wild-type and Arctic-type Aβ_40_ and decrease the rates of structural conversion and fibril formation. However, the underlying mechanism for the increase of structural stability as a result of the alanine substitutions remained elusive. In this study, we apply nuclear magnetic resonance and circular dichroism spectroscopies to characterize the Aβ_40_ structure, demonstrating that L17A/F19A substitutions can augment the α-helicity of the residues located in the α/β-discordant segment (resides 15 to 23) of both wild-type and Arctic-type Aβ_40_. These results provide a structural basis to link the α-helicity of the α/β-discordant segment with the conformational conversion propensity of Aβ.

## Introduction

β-amyloid peptide (Aβ), consisting of 39–42 residues, is derived from the proteolytic product of a type I transmembrane glycoprotein called β-amyloid precursor protein (AβPP). Alzheimer’s disease (AD) is highly associated with Aβ aggregation. The molecular mechanism for Aβ aggregation remained unclear. The conformational change is occurred during the Aβ aggregation process. Recently, we have applied small Aβ-binding molecules to probe the aggregation mechanism of Aβ_40_. The results indicated that the small molecules targeted to interact with the hydrophobic central region (L_17_VFFA_21_) of wild-type Aβ_40_ can stabilize its conformation and block the formation of amyloid fibril [1]. Our previous structural characterizations by using nuclear magnetic resonance (NMR) spectroscopy, equilibrium denaturation and site-directed mutagenesis have also demonstrated that the secondary structure of the hydrophobic central region of Aβ was more prone to unfold than that of the rest of the regions in Aβ. Double replacements of the two residues (L17/F19) in this region by Alanine could block conformational changes and reduce cytotoxicity of wild-type Aβ_40_ [2]. Arctic-type Aβ_40_ (Aβ_40_(E22G)), causing one of the familial Alzheimer’s diseases (FAD), has been known to show a stronger aggregative ability than wild-type Aβ_40_. By introducing double replacements (L17A/F19A) into Arctic-type Aβ_40_, similar effects were also observed for this FAD-linked Aβ_40_ variant [3], suggesting that the conformational stability in the hydrophobic central region of Aβ plays a critical role in the conformational conversion tendency of Aβ. There were also many studies of the hydrophobic central region of Aβ, which supported the view that the hydrophobic central region is highly correlated with Aβ aggregation and can be utilized for designing inhibitors [4–11].

The molecular mechanism for L17A/F19A substitutions to stabilize the conformations of the Aβ peptides remained unknown. By using circular dichroism (CD) spectroscopy, Johannson and coworkers showed that the overall α-helical propensity of Aβ_12–28_ was increased after V18A/F19A/F20A replacements. This triple Ala-substituted Aβ_12–28_ was predicted to form an α-helix in the region of the α/β-discordant segment, suggesting that V18A/F19A/F20A replacements abolished the discordance, resulting in inhibition of fibril formation of Aβ_12–28_ [12]. It is likely that double replacements (L17A/F19A) could enhance the α-helicity in this region, in turn leading to an increase of their structural stability. However, it lacks the structural evidence to support this inference.

To demonstrate this hypothetic mechanism, we characterized the effects of L17A/F19A substitutions on the structures of wild-type and Arctic-type Aβ_40_ by using nuclear magnetic resonance (NMR) and CD spectroscopies. Stable isotope labeled Aβ_40_, Aβ_40_(E22G), Aβ_40_(L17A/F19A) and Aβ_40_(L17A/F19A/E22G) were prepared in this study for NMR structural characterization. The propensity of secondary structure in these peptides were characterized in a residue-specific manner. An augmentation of α-helicity in the α/β-discordant segment was observed for both wild-type and Arctic-type Aβ_40_ after L17A/F19A substitutions. These results may explain the reason why L17A/F19A substitutions increase the conformational stability of these two Aβ_40_ peptides.

## Materials and Methods

### Sample Preparation

All Aβ peptides used in this study were produced using the protocols as described previously [13]. The cDNA of wild-type Aβ_40_ was served as a template for the site-directed mutagenesis to create the cDNA of Aβ_40_ mutants. All procedures followed the methods as described by manufacturer (QuikChange Lightning, Stratagene). Preparation of the stable isotope-labeled (^13^C/^15^N and ^15^N) Aβ peptides followed the methods as described in [2, 14].

### CD Spectroscopy

All purified Aβ peptides that have been verified by mass spectrometry were pretreated with 100% TFE (trifluoroethanol) and then dried by nitrogen gas. The dried Aβ molecule was dissolved in 10 mM K_2_HPO_4_/KH_2_PO_4_ buffer solution containing 100 mM SDS-d_25_ (sodium dodecyl sulfate-d_25_) (pH 6.0). All Aβ molecules (50 μM) were used for analysis by CD spectroscopy (Aviv410 spectropolarimeter, Aviv Biomedical, Inc., Lakewood, NJ USA). The spectra were collected at 296 K and the wavelengths were scanned from 190 to 260 nm in 0.2-nm increments. The measurement was carried out three times. The secondary structure contents of Aβ were estimated by using CDNN program [15, 16].

### NMR Spectroscopy

For NMR studies, the dried Aβ molecule was dissolved in 10 mM K_2_HPO_4_/KH_2_PO_4_ buffer solution containing 100 mM SDS-d_25_, 10% (v/v) D_2_O/H_2_O, 0.02% NaN_3_ and the internal chemical shift standard, TSP (3-(trimethylsilyl)propionic-2,2,3,3,-d_4_ acid) (pH 6.0). The NMR data processing and the determination of backbone chemical shifts followed the methods as described in [2, 14].

## Results

**Fig 1:**
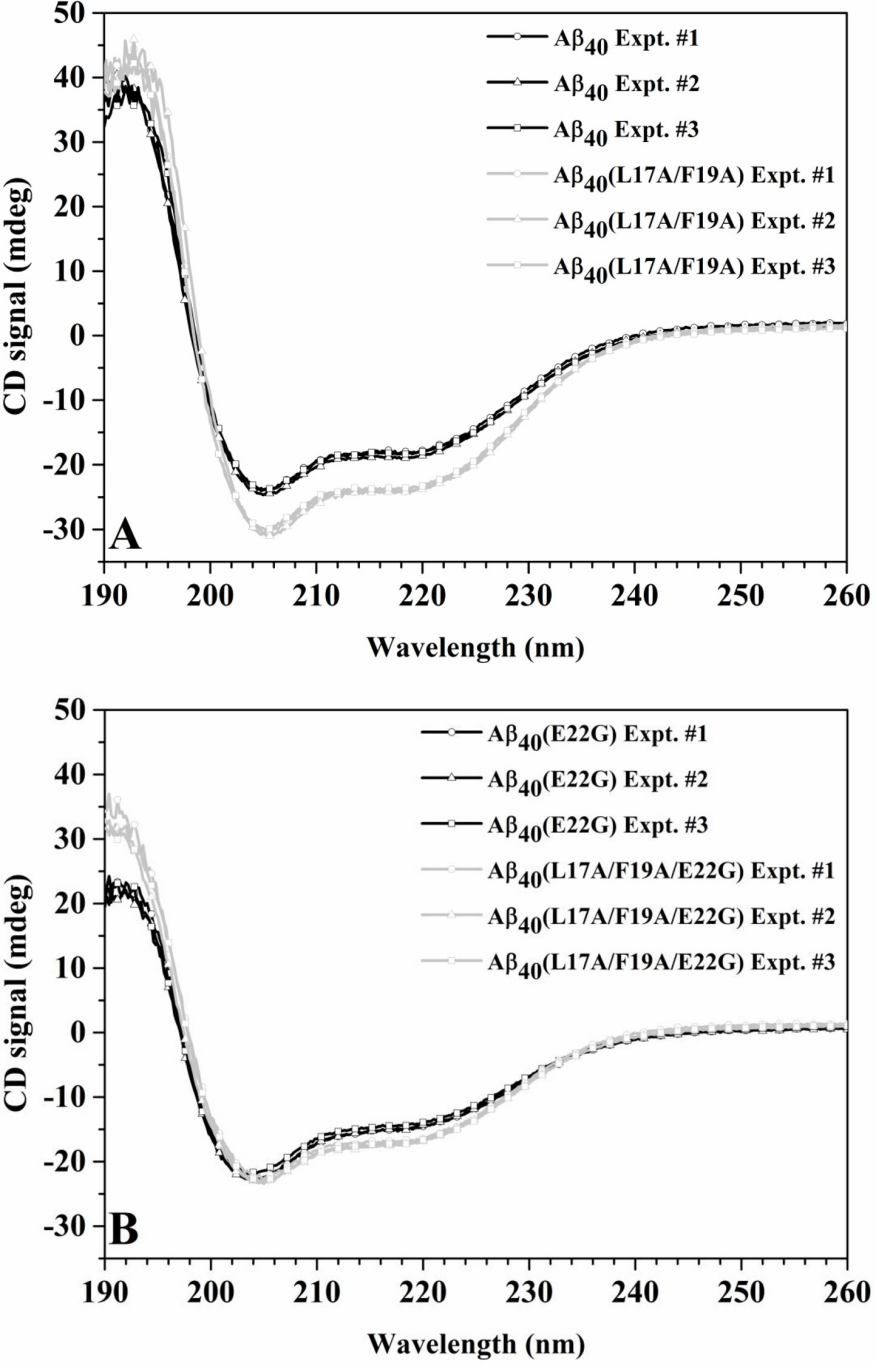
CD spectra of wild-type Aβ_40_ and Arctic Aβ_40_ variant in 100 mM SDS solution. (A) Superimposition of CD spectra of Aβ_40_(L17A/F19A) (light grey) and wild-type Aβ_40_ (black) in 100 mM SDS solution. (B) Superimposition of CD spectra of Aβ_40_(L17A/F19A/E22G) (light grey) and Aβ_40_(E22G) (black) in 100 mM SDS solution.

We first characterized the effect of L17A/F19A substitutions on the secondary structure contents of wild-type and Arctic-type Aβ_40_ by using CD spectroscopy. The CD spectra of wild-type Aβ_40_ and Aβ_40_(L17A/F19A) in SDS solution were shown in Fig 1A. Both peptides exhibited two major bands in their CD spectra with minima at 206 and 220 nm (negative ellipticities at 206 and 220 nm). This spectral pattern is an indicative of α-helical structure for both peptides. In addition, the intensities at 206 and 220 nm were more negative for Aβ_40_(L17A/F19A) than for wild-type Aβ_40_, indicating that the α-helicity of Aβ_40_(L17A/F19A) is higher than that of wild-type Aβ_40_. The effect of L17A/F19A substitutions on the CD spectrum of Arctic-type Aβ_40_ in SDS solution was shown in Fig 1B. The spectral patterns shown in Fig 1B were similar to those in Fig 1A. The intensities at 206 and 220 nm were a little bit more negative for Aβ_40_(L17A/F19A/E22G) than for Aβ_40_(E22G), suggesting that the conformation of Aβ_40_(L17A/F19A/E22G) contains higher α-helicity than that of Aβ_40_(E22G). We also used the CDNN program [15, 16] to analyze the CD spectra to estimate the secondary structure contents of wild-type Aβ_40_, Aβ_40_(L17A/F19A), Aβ_40_(E22G) and Aβ_40_(L17A/F19A/E22G). The results were shown in Table 1. Both double Ala-substituted Aβ peptides displayed higher α-helix and lower β-strand contents than their native forms. These findings indicated that double replacements (L17A/F19A) increased the α-helicity of both wild-type and Arctic-type Aβ_40_. Since CD spectra can only provide information of overall structural differences, the region of these double Ala-substituted peptides for the increases of α-helical contents remained characterized.

**Table 1:** The secondary structure contents estimated from the CD spectra of Aβ peptides.

	Aβ_40_	Aβ_40_(L17A/F19A)	Aβ_40_(E22G)	Aβ_40_(L17A/F19A/E22G)
Helix	32.10 ± 0.26%	37.97 ± 0.91%	26.13 ± 0.35%	28.90 ± 0.26%
Antiparallel	13.47 ± 0.45%	9.03 ± 0.81%	24.10 ± 0.78%	18.50 ± 0.69%
Parallel	7.60 ± 0.10%	6.40 ±0.10%	8.70 ± 0.10%	8.13 ± 0.06%
Beta-turn	18.00 ± 0.10%	17.10 ± 0.17%	19.43 ± 0.12%	18.73 ± 0.06%
Random coil	26.13 ± 0.29%	21.67 ± 0.21%	28.63 ± 0.40%	27.37 ± 0.21%

The secondary structure contents were shown as the average **±** standard deviation from three individual CD experiments. Statistical analysis of the secondary structure contents was done by using GraphPad Prism Software (GraphPad Software, La Jolla, CA, USA). For helix, antiparallel and random coil values, the difference between Aβ_40_ and Aβ_40_(L17A/F19A) by one-way ANOVA analysis with a Tukey Multiple Comparison Test showed statistical significance (*p* < 0.05). There is no statistically significant difference for parallel and beta-turn values. The difference between Aβ_40_(E22G) and Aβ_40_(L17A/F19A/E22G) also show statistical significance for helix, antiparallel and random coil values, and no statistical significance for parallel and beta-turn values.

**Fig 2:**
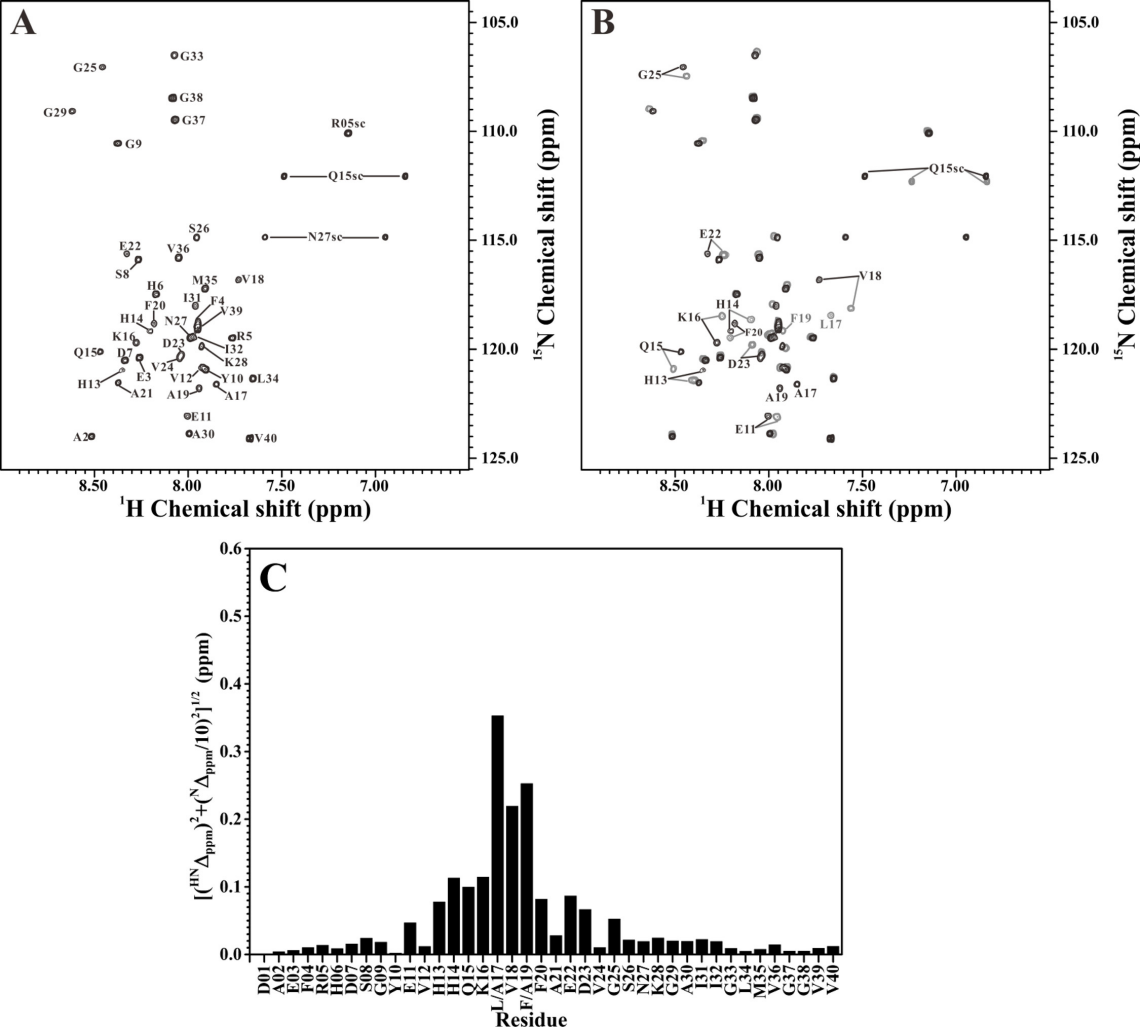
Comparison of the 2D ^1^H-^15^N-HSQC spectra of wild-type Aβ_40_ and Aβ_40_(L17A/F19A). (A) 2D ^1^H-^15^N-HSQC spectrum of Aβ_40_(L17A/F19A) in 100 mM SDS solution. (B) Superimposition of 2D ^1^H-^15^N-HSQC spectra of Aβ_40_(L17A/F19A) (black) and wild-type Aβ_40_ (light grey) in 100 mM SDS solution. Residues with noticeable chemical shift changes were labeled. (C) The effect of L17A/F19A replacements on the backbone amide resonances of wild-type Aβ_40_. The weighted chemical shift differences ([(^HN^Δ_ppm_)^2^+ (^N^Δ_ppm_/10)^2^]^1/2^) were plotted as a function of residue number. ^HN^Δ_ppm_ and ^N^Δ_ppm_ were the ^1^H^N^ and ^15^N chemical shift differences between wild-type Aβ_40_ and Aβ_40_(L17A/F19A), respectively.

**Fig 3:**
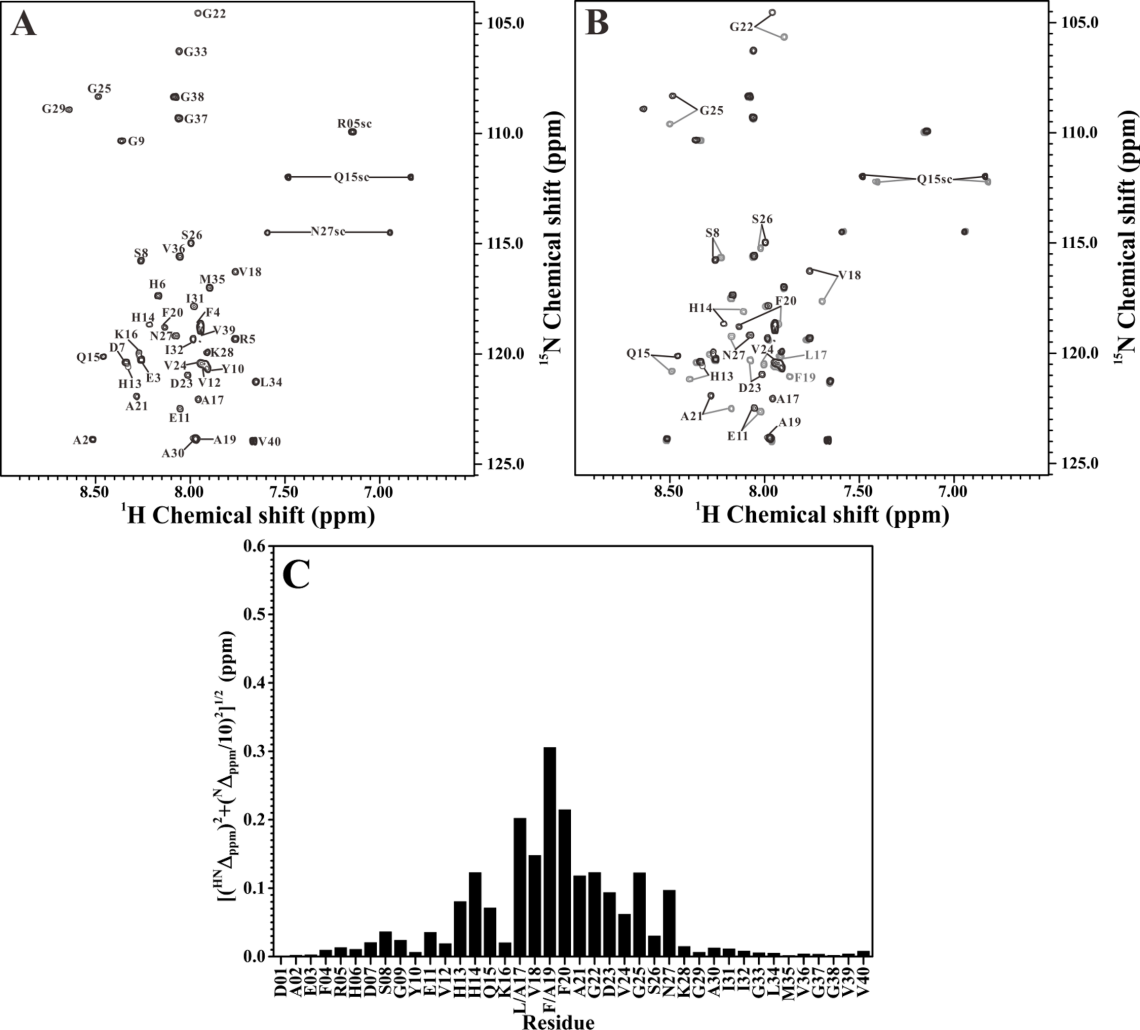
Comparison of the 2D ^1^H-^15^N-HSQC spectra of Aβ_40_(E22G) and Aβ_40_(L17A/F19A/E22G). (A) 2D ^1^H-^15^N-HSQC spectrum of Aβ_40_(L17A/F19A/E22G) in 100 mM SDS solution. (B) Superimposition of 2D ^1^H-^15^N-HSQC spectra of Aβ_40_(L17A/F19A/E22G) (black) and Aβ_40_(E22G) (light grey) in 100 mM SDS solution. Residues with noticeable chemical shift changes were labeled. (C) The effect of L17A/F19A replacements on the backbone resonances of Aβ_40_(E22G). The weighted chemical shift differences ([(^HN^Δ_ppm_)^2^+(^N^Δ_ppm_/10)^2^]^1/2^) were plotted as a function of residue number. ^HN^Δ_ppm_ and ^N^Δ_ppm_ were the ^1^H^N^ and ^15^N chemical shift differences between Aβ_40_(E22G) and Aβ_40_(L17A/F19A/E22G), respectively.

We next applied NMR spectroscopy to analyze the structural differences between the double Ala-substituted Aβ peptides and their native forms. Fig 2A showed the 2D ^1^H-^15^N-HSQC spectrum of ^15^N-labeled Aβ_40_(L17A/F19A) in SDS solution. The assigned residues were indicated in the figure. The effect of L17A/F19A substitutions on the 2D ^1^H-^15^N-HSQC spectrum of wild-type Aβ_40_ was shown in Fig 2B. It is apparent that some amide proton and nitrogen cross-peaks of wild-type Aβ_40_ displayed noticeable chemical shift changes after the replacements of L17 and F19 with alanines. Compared to the previously assigned backbone resonances of wild-type Aβ_40_ [14], these cross-peaks were identified as E11, H13-K16, V18, F20, E22, D23 and G25 (excluding L17 and F19). Fig 2C showed the backbone amide chemical shift differences between wild-type Aβ_40_ and Aβ_40_(L17A/F19A). Most of these residues that showed noticeable chemical shift changes after L17A/F19A substitutions were located within the α/β-discordant segment of wild-type Aβ_40_. Fig 3A and 3B showed the 2D ^1^H-^15^N-HSQC spectrum of ^15^N-labeled Aβ_40_(L17A/F19A/E22G) without and with superimposition of the 2D ^1^H-^15^N-HSQC spectrum of ^15^N-labeled Aβ_40_(E22G), respectively. Comparison of the 2D ^1^H-^15^N-HSQC spectra for Aβ_40_(E22G) and Aβ_40_(L17A/F19A/E22G) in Fig 3B showed that noticeable chemical shift changes also occurred on some amide proton and nitrogen cross-peaks of Aβ_40_(E22G) after L17A/F19A substitutions. Cross-peaks that displayed noticeable chemical shift changes were identified as S8, E11, H13-Q15, V18, F20-N27 (excluding L17 and F19), as compared to the previously assigned backbone resonances of Aβ_40_(E22G) [14]. The backbone amide chemical shift differences between Aβ_40_(E22G) and Aβ_40_(L17A/F19A/E22G) were shown in Fig 3C. The majority of these residues that displayed noticeable chemical shift changes after L17A/F19A substitutions were also located within the α/β-discordant segment of Aβ_40_(E22G). This effect induced by L17A/F19A substitutions was very similar to that observed in wild-type Aβ_40_. Our NMR characterizations suggested that the increases of α-helicity observed by CD spectroscopy might mainly occur at the residues in the α/β-discordant segment of these double Ala-substituted peptides.

**Fig 4:**
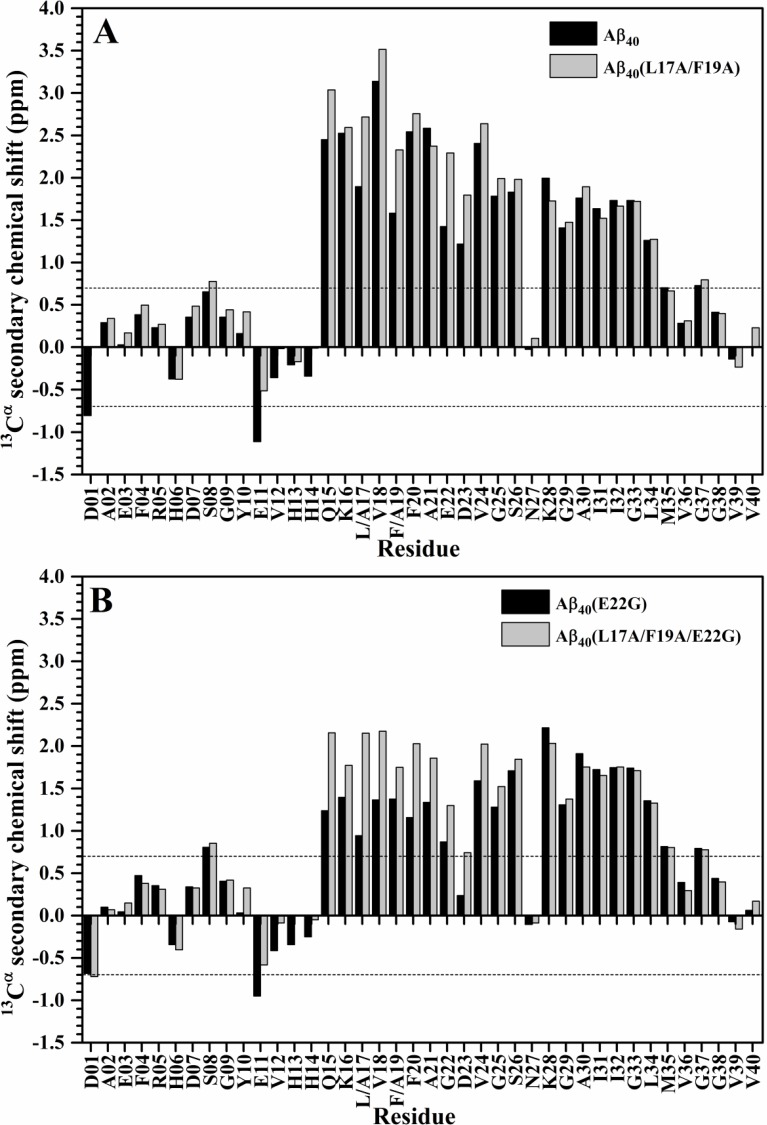
Comparison of ^13^C^α^ secondary chemical shifts of double Ala-substituted Aβ peptides and their native forms. (A) The plots of ^13^C^α^ secondary chemical shifts of Aβ_40_(L17A/F19A) (light grey) and wild-type Aβ_40_ (black) as a function of residue. (B) The plots of ^13^C^α^ secondary chemical shifts of Aβ_40_(L17A/F19A/E22G) (light grey) and Aβ_40_(E22G) (black) as a function of residue.

Since sequence effect may also induce chemical shift changes of amide proton and nitrogen cross-peaks, the chemical shift changes resulting from L17A/F19A substitutions might not solely come from the alteration of α-helical propensity. To verify whether the increases of α-helical propensities occurred at the residues in the α/β-discordant segment of these double Ala-substituted peptides, we further analyzed the ^13^C^α^ secondary chemical shifts of all mutant Aβ_40_ peptides, and compared them with those of their native forms [14]. It has been reported that the ^13^C^α^ chemical shift is sensitive to protein backbone structure [17]. The ^13^C^α^ secondary chemical shift which is defined as the deviation of the observed ^13^C^α^ chemical shift of an amino acid residue from its ^13^C^α^ chemical shift in a random coil conformation has been used as a measure of secondary structure propensity [18]. For an amino acid residue in an α-helical conformation, it has an average ^13^C^α^ secondary chemical shift of 3.09 ± 1.0 ppm [17]. This value was used to estimate the percent α-helicity (% α-helicity) of an amino acid residue as well. Weinstock et al. showed that the percent α-helicity of an amino acid residue calculated from structure is quantitatively in agreement with that calculated from ^13^C^α^ secondary chemical shift, demonstrating that ^13^C^α^ secondary chemical shift is correlated to percent α-helicity [19]. The more positive ^13^C^α^ secondary chemical shifts represents the higher percent α-helicity. According to this correlation, we obtained that the replacements of L17 and F19 with alanines mainly augment the α-helicity of residues Q15, V18, F20 and E22-G25 (excluding L17 and F19) of wild-type Aβ_40_ in SDS solution. Fig 4A showed that the ^13^C^α^ secondary chemical shifts of these residues were significantly more positive for Aβ_40_(L17A/F19A) than for wild-type Aβ_40_. The similar phenomenon was also observed in residues Q15-G25 of Aβ_40_(L17A/F19A/E22G) and Aβ_40_(E22G), as shown in Fig 4B, suggesting that residues Q15-G25 (excluding L17 and F19) of Aβ_40_(L17A/F19A/E22G) adopted a higher α-helicity than those of Aβ_40_(E22G).

Previously, Wishart et al. used chemical shit index (CSI) to determine secondary structure. An α-helix is identified as a group of three or more consecutive amino acid residues whose ^13^C^α^ secondary chemical shift were greater than 0.7 ppm [20]. It can be seen that the ^13^C^α^ secondary chemical shift of D23 in Aβ_40_(E22G) was much less than 0.7 ppm, however, the ^13^C^α^ secondary chemical shift of D23 in Aβ_40_(L17A/F19A/E22G) was greater than 0.7 ppm. This result suggested that the lost α-helicity at D23 of Arctic-type Aβ_40_ [14] might be restored after L17A/F19A substitutions. The results of ^13^C^α^ secondary chemical shift analysis further supported the fact that L17A/F19A substitutions mainly increased the α-helicity of the α/β-discordant segment in both wild-type and Arctic-type Aβ_40_.

## Discussion

The structure of Aβ is dependent on the environments in which Aβ exists. It may adopt either random coil or α-helical conformation in different environments, such as in aqueous solution and SDS solution [21, 22]. The reason for using aqueous SDS solution to probe the α-helical propensity of Aβ and the relevance of this environment to biological systems have been discussed from a structural perspective in our previous publication [14]. In fibrillar form, Aβ mainly adopts β-strand conformation [23, 24]. The structure of nascent wild-type Aβ_40_ residing in cellular membranes before unfolding remained unclear. However, the structure of C99, the transmembrane C-terminal domain of AβPP (AβPP_672-770_), in lipid environments has been reported [25, 26]. The structure of wild-type Aβ_40_ in SDS solution resembles the structure of the Aβ_40_ domain (AβPP_672-711_) in C99 in lipid environments and can be considered as the initial structure of wild-type Aβ_40_ in cellular membranes. From a structural perspective, these suggested that Aβ peptides have to undergo a structural conversion from α-helix to β-strand during the aggregation process. From a thermodynamical point of view, the activation energy of structural conversion from α-helix to β-strand is closely related to the secondary structure propensity. For instance, a higher α-helical propensity would result in a higher activation energy for structural conversion from α-helix to β-strand. Thus, the secondary structure propensity of Aβ would be one key factor in governing its structural conversion tendency. Previously, Johannson and coworkers applied an *in silico* approach to predict amyloid fibril-forming proteins and proposed that these proteins contained an α/β-discordant sequence which is expected to form a β-strand but displays an α-helical structure in some environments and supposed to be prone to undergo a conformational transition from α-helix to β-strand. Aβ peptide was predicted to contain an α/β-discordant sequence located in the region of residues 16–23 [27], suggesting that the tendency for structural conversion of Aβ might be mainly governed by the secondary structure propensity of its α/β-discordant segment. It is likely that the structural transition occurred in the α/β-discordant segment prompted the aggregation cascade of Aβ peptide. Thus, any factor that varies the propensity of secondary structure in the α/β-discordant segment of Aβ would affect its structural conversion tendency, resulting in an alteration of Aβ aggregation propensity, such as mutation occurred in the α/β-discordant segment of Aβ. This conclusion has been confirmed by our recent studies [14]. We have demonstrated that Arctic mutation accelerates Aβ aggregation in SDS through diminishing the α-helicity of residues 15–25.

**Fig 5:**
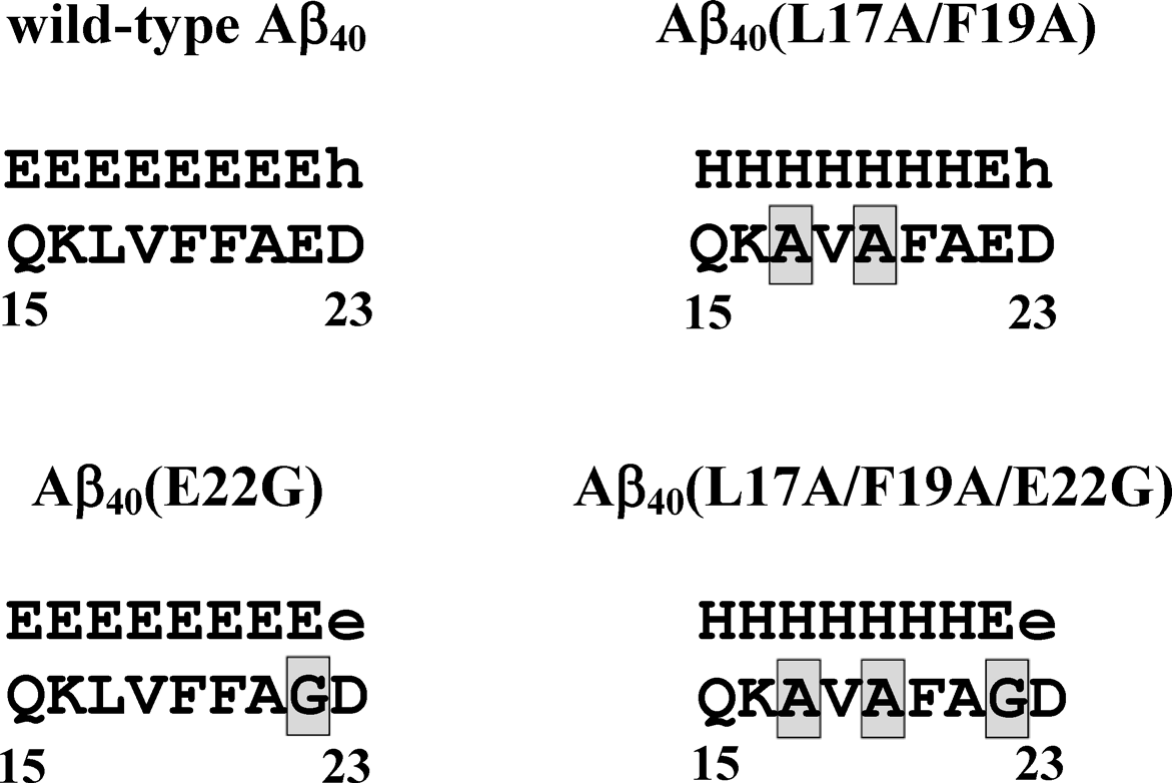
The predicted secondary structures of the α/β-discordant segments of double Ala-substituted Aβ peptides and their native forms. The secondary structure (upper row) for each amino acid residue was obtained by using the propensity-based prediction as described in Fig 2 caption of ref. 27. Adopting the notation used in Fig 2 caption of ref. 27, we denote the β-strands predicted with high and low probability by the symbols E and e, respectively. The symbols H and h were used for denoting the α-helical structures predicted with high and low probability, respectively.

We previously found that the secondary structure of Aβ has relatively unstable residues, L17 and F19, in the α/β-discordant segment. L17A/F19A substitutions may reduce the rates of structural conversions and fibril formation of both wild-type [2] and Arctic-type Aβ_40_ [3]. The result of *in silico* prediction also suggested that L17A/F19A substitutions may alter the propensity of the secondary structure of the α/β-discordant segment for both wild-type and Arctic-type Aβ_40_. Fig 5 showed the secondary structures of the α/β-discordant segments obtained by using the propensity-based prediction [27]. In the present study, our data show that L17A/F19A substitutions can augment the α-helicity of the α/β-discordant segment for both peptides, confirming that an increase of the α-helical propensity of the α/β-discordant segment can stabilize the conformation and reduce the structural conversion tendency, in turn leading to a reduction of Aβ aggregation propensity.

A nucleation-dependent polymerization model has been pointed out to illustrate this complicated process for Aβ aggregated into amyloid fibril [28]. According to this model, the aggregation process of Aβ involves conformational changes and self-assembly. In addition to the intrinsic structural propensity of Aβ, the intramolecular and/or intermolecular interactions of Aβ also play an important role in the aggregation tendency of Aβ. In a real biological system, the interactions between Aβ and its local environments would affect its aggregation propensity. Moreover, the conformation of Aβ may influence these interactions as well. Mutation in the α/β-discordant segment of Aβ might alter not only the structural propensity but also these interactions. The effects of mutations on these two factors are profound. Either one factor could increase or reduce the structural conversion tendency of Aβ. The joint contribution of these two factors to the conformational conversion tendency of Aβ would result in a modulation of Aβ aggregation propensity. In the previous study [14], we found that Arctic mutation decreased the α-helical propensity of the α/β-discordant segment, leading to in an increase of the structural conversion tendency of Aβ. In this study, we obtain that L17/F19A substitutions exhibits an opposite effect on the structural propensity and structural conversion tendency of Aβ. These mutation studies provided the information about the role of the structural propensity of the α/β-discordant segment in Aβ aggregation propensity. However, the role of the interactions in Aβ aggregation propensity remains characterized. The α/β-discordant segment of Aβ covered residues 16–23. Besides the Arctic mutation, several FAD-linked AβPP mutations which promote α-helix-to-β-strand conversion and fibril formation [29, 30] were also located in this region. Whether other FAD-linked AβPP mutations have similar effects to Arctic mutation on the structural propensity of the α/β-discordant segment remains unknown. The effects of L17/F19A substitutions on the structural and aggregative propensities of other FAD-linked Aβ mutants need to be investigated. Study of the effect of these mutations on the structural and aggregative propensities of Aβ may help us gain more insight into the molecular mechanism of Aβ aggregation from a structural point of view.

## Abbreviations

Aβ: β-amyloid peptide
AβPP: β-amyloid precursor protein
AD: Alzheimer’s disease
CD: circular dichroism
CSI: chemical shift index
FAD: familial Alzheimer’s disease
NMR: nuclear magnetic resonance
SDS: sodium dodecyl sulfate
TFE: trifluoroethanol
